# Technical note: Preprocedural PET/CT guidance for fine needle aspiration cytology of a lung mass

**DOI:** 10.4103/0971-3026.38509

**Published:** 2008-02

**Authors:** MJ Govindarajan, Arjun Kalyanpur, KR Nagaraj, H Ravikumar, KG Kallur, PS Sridhar

**Affiliations:** Bangalore Institute of Oncology and Health Care Global and Teleradiology Solutions, Bangalore, India; 1 Teleradiology Solutions, Bangalore, India; 2 Department of Nuclear Medicine, Bangalore Institute of Oncology and Health Care Global, Bangalore, India; 3 Bangalore Institute of Oncology and Health Care Global, Bangalore, India

When a lung nodule is diagnosed, it is necessary to differentiate a benign lesion from one that is indeterminate/aggressive. Due to the increased possibility of malignancy in patients above the age of 35, with nodules >10 mm in size, immediate, often invasive, workup is required, including contrast-enhanced dynamic CT, positron emission tomography (PET), or biopsy.[1] However, at times, optimal localization of the fine needle aspiration cytology (FNAC)/biopsy site may not be possible as the more easily accessible areas may not necessarily be metabolically active. PET/CT may identify metabolically active areas within the mass and help to optimize the diagnostic tissue yield - PET providing physiological information and CT providing the anatomical details. We present one such case, where a prior CT-guided FNAC was unsuccessful but a subsequent PET/CT allowed proper guidance for correct localization of the FNAC site.

## Case Report

**Figure 1:**
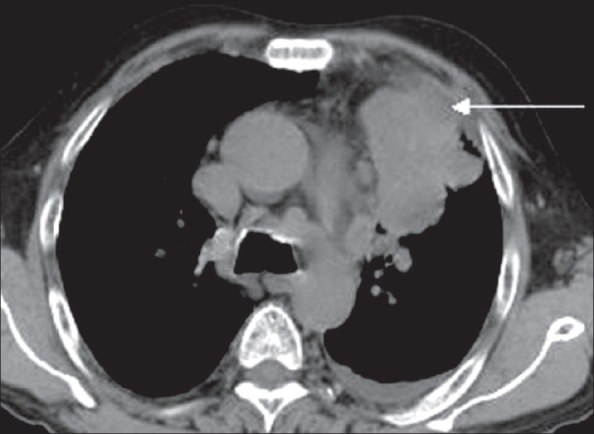
Plain CT scan shows a peripherally located left upper lobe mass with heterogeneous density, demonstrating a large area abutting the pleura (arrow), which is typically the preferred site for a biopsy

**Figure 2:**
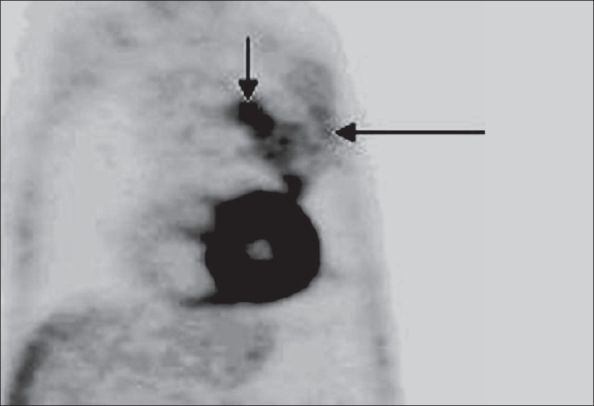
Coronal F18 FDG PET image shows that the peripheral and inferior portions are relatively less metabolically active (long arrow) as compared to the superomedial portion (short arrow), which shows the maximum metabolic activity

**Figure 3:**
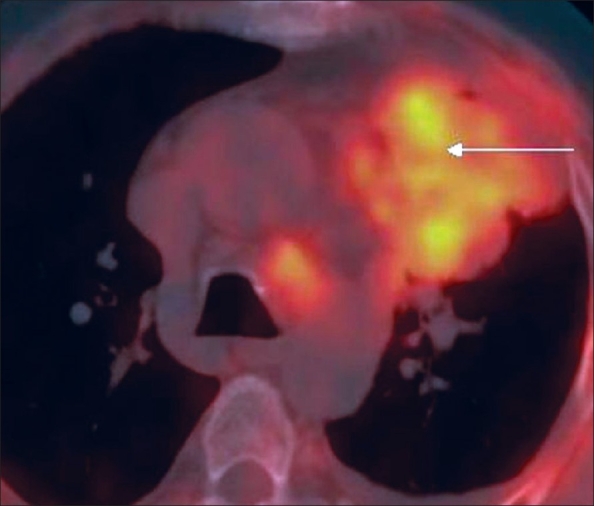
Fused PET/CT axial image gives morphological and physiological information, allowing proper planning of the FNAC site (arrow)

**Figure 4:**
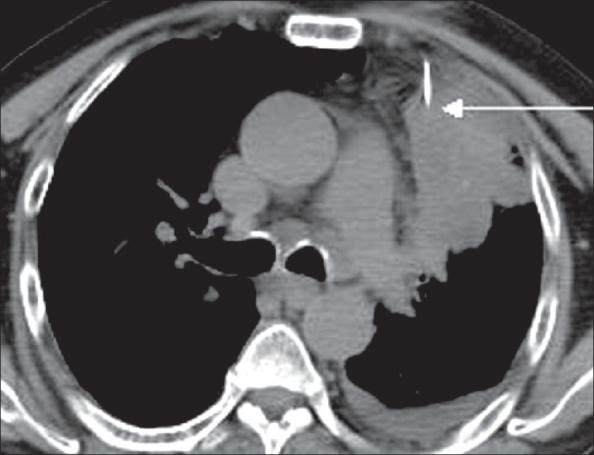
Plain CT of the chest shows the needle in position (arrow) in the area of maximum metabolic activity

A 60-year-old man without a history of smoking, presented with unexplained weight loss and cough with chest pain. A chest radiograph revealed a left mid-zone opacity. CT scan demonstrated a relatively large, predominantly pleural-based mass in the left upper lobe [Figure 1]. The patient had undergone a percutaneous CT-guided FNAC a few days before but the yield had not been adequate for a confident interpretation. PET/CT was performed using F18 FDG on a 16-slice multi-detector CT scanner (Discovery STE, GE). The PET study showed that the peripheral and inferior portions of the lesion, which were most easily accessible for biopsy, were only minimally metabolically active, while the inner and upper portions of the mass were more active [Figure 2]. The fused PET/CT images showed the exact area where the FNAC had to be planned [Figure 3]. FNAC was then performed [Figure 4] and the histopathologic diagnosis was pleomorphic sarcomatoid carcinoma.

## Discussion

The importance of limiting repeat procedures cannot be overemphasized. It is especially important in cases of lung masses, where the chance of significant pneumothorax increases with repeated attempts.[2] In the case of lung lesions, contrast-enhanced CT scans may be able to differentiate between the mass and the adjacent atelectatic lung. However, it is sometimes difficult to do so, especially with peripheral lesions.[3,4] In our case, intravenous contrast was not administered as the patient had already had a recent diagnostic CT scan, followed by an attempted guided FNAC. The central portions of the mass are often necrotic and a good FNAC/biopsy should avoid these areas. PET scan with FDG helps in delineating metabolically active areas and, along with a CT scan performed at the same time (PET/CT), can help localize the area most likely to give a diagnostic yield during an FNAC/biopsy.

In our case, the peripheral and inferior portions of the lesion, which were more easily accessible percutaneously, were minimally metabolically active and this was probably the reason for the lack of success with the prior CT-guided FNAC.

The literature on the use of PET/CT for interventional guidance is sparse. A recent *ex vivo* study in pigs showed PET/CT to be more accurate than CT alone for image-guided interventions in liver lesions.[5] PET/CT-guided percutaneous puncture of an infected cyst in a patient with autosomal dominant polycystic kidney disease has also been reported;[6] the exact site/infected cyst could be demonstrated by the PET study. PET/CT has been used for the localization of the electrode tip during radiofrequency ablation of hepatic metastases.[7] PET/CT is also useful in previously treated lesions, where it can differentiate between viable and necrotic areas and guide biopsy/intervention if required.[8]

Logically, with the incorporation of morphological details from CT and MRI and functional details from PET, the ability to guide intervention should improve considerably[9,10] and, hopefully, properly designed studies may confirm this in the future.
